# Randomized phase II study of pemetrexed/cisplatin with or without axitinib for non-squamous non-small-cell lung cancer

**DOI:** 10.1186/1471-2407-14-290

**Published:** 2014-04-25

**Authors:** Chandra P Belani, Nobuyuki Yamamoto, Igor M Bondarenko, Artem Poltoratskiy, Silvia Novello, Jie Tang, Paul Bycott, Andreas G Niethammer, Antonella Ingrosso, Sinil Kim, Giorgio V Scagliotti

**Affiliations:** 1 Penn State Milton S. Hershey Medical Center, Penn State Hershey Cancer Institute, Hershey, PA, USA; 2 Third Department of Internal Medicine, Wakayama Medical University, Wakayama, Japan; 3 Department of Oncology and Medical Radiology, Dnipropetrovsk State Medical Academy, Dnipropetrovsk, Ukraine; 4 Department of Thoracic Oncology, St. Petersburg Medical University, St. Petersburg, Russia; 5 Department of Oncology, San Luigi Hospital, University of Torino, Torino, Italy; 6 Oncology Business Unit, Pfizer Inc, New York, NY, USA; 7 Clinical Development, Pfizer Oncology, San Diego, CA, USA; 8 Clinical Development, Pfizer Oncology, Milano, Italy

**Keywords:** Axitinib, Pemetrexed, Cisplatin, Non-squamous, NSCLC

## Abstract

**Background:**

The efficacy and safety of axitinib, a potent and selective second-generation inhibitor of vascular endothelial growth factor receptors 1, 2, and 3 in combination with pemetrexed and cisplatin was evaluated in patients with advanced non-squamous non–small-cell lung cancer (NSCLC).

**Methods:**

Overall, 170 patients were randomly assigned to receive axitinib at a starting dose of 5-mg twice daily continuously plus pemetrexed 500 mg/m^2^ and cisplatin 75 mg/m^2^ on day 1 of up to six 21-day cycles (arm I); axitinib on days 2 through 19 of each cycle plus pemetrexed/cisplatin (arm II); or pemetrexed/cisplatin alone (arm III). The primary endpoint was progression-free survival (PFS).

**Results:**

Median PFS was 8.0, 7.9, and 7.1 months in arms I, II, and III, respectively (hazard ratio: arms I vs. III, 0.89 [*P* = 0.36] and arms II vs. III, 1.02 [*P* = 0.54]). Median overall survival was 17.0 months (arm I), 14.7 months (arm II), and 15.9 months (arm III). Objective response rates (ORRs) for axitinib-containing arms were 45.5% (arm I) and 39.7% (arm II) compared with 26.3% for pemetrexed/cisplatin alone (arm III). Gastrointestinal disorders and fatigue were frequently reported across all treatment arms. The most common all-causality grade ≥3 adverse events were hypertension in axitinib-containing arms (20% and 17%, arms I and II, respectively) and fatigue with pemetrexed/cisplatin alone (16%).

**Conclusion:**

Axitinib in combination with pemetrexed/cisplatin was generally well tolerated. Axitinib combinations resulted in non-significant differences in PFS and numerically higher ORR compared with chemotherapy alone in advanced NSCLC.

**Trial registration:**

ClinicalTrials.gov: NCT00768755 (October 7, 2008).

## Background

Currently, the majority of patients with non–small-cell lung cancer (NSCLC) present with inoperable, locally advanced (stage IIIB) or metastatic (stage IV) disease for which no curative therapy is available, and the 5-year survival rate has remained ≤5% for the last few decades [1,2]. In patients with advanced or metastatic NSCLC without certain cytogenetic abnormalities (e.g. epidermal growth factor receptor [*EGFR*] mutations, anaplastic lymphoma kinase [ALK] translocations), platinum-based doublet chemotherapy remains the standard of care, albeit with modest efficacy [3], necessitating the search for additional treatment approaches to improve clinical outcomes. Because angiogenesis plays a critical role in tumor survival, growth, and metastasis, inhibition of the key angiogenesis pathway mediated via vascular endothelial growth factor (VEGF)/VEGF receptor signaling, either at the ligand level (e.g. bevacizumab) or at the receptor level (e.g. the tyrosine kinase inhibitors [TKIs] sorafenib, sunitinib, pazopanib, or axitinib, among many others), has been intensively evaluated in advanced NSCLC [4,5]. Addition of bevacizumab to paclitaxel and carboplatin was shown to improve overall survival (OS) compared with chemotherapy alone in patients with advanced non-squamous NSCLC, providing evidence of therapeutic benefit in combining an antiangiogenic agent with chemotherapy [6]. However, the extent of survival gained from the addition of bevacizumab to chemotherapy may still be considered modest.

Axitinib is a potent and selective second-generation inhibitor of VEGF receptors 1, 2, and 3 [7] approved in the United States, European Union, Japan, and elsewhere for the treatment of advanced renal cell carcinoma after failure of one prior systemic therapy. Axitinib also showed promising single-agent activity with an acceptable safety profile in an open-label, single-arm, phase II trial in advanced NSCLC [8]. In treatment-naïve (*n* = 9) and previously treated *(n* = 23) patients with advanced NSCLC, objective response rate (ORR) was 9%, with median progression-free survival (PFS) and OS of 4.9 and 14.8 months, respectively. Common adverse events (AEs) included fatigue, anorexia, diarrhea, nausea, and hypertension. Axitinib was also generally well tolerated when administered in combination with standard chemotherapy in patients with advanced solid tumors, including NSCLC [9], which is the basis for the current study.

This study was undertaken to evaluate the efficacy and safety of combining axitinib with the pemetrexed/cisplatin regimen compared with pemetrexed/cisplatin alone in patients with advanced or recurrent non-squamous NSCLC. The choice of backbone chemotherapy was based on a large prospective phase III trial [10] that demonstrated OS superiority with better tolerability of pemetrexed/cisplatin over that of cisplatin/gemcitabine in NSCLC. In addition, axitinib was administered in two different dosing schedules (continuously vs. intermittently) to investigate whether a 2-day break in axitinib dosing just prior to chemotherapy administration would improve efficacy.

## Methods

### Patients

Patients aged 18 years and older (≥20 years in Japan) with histologically or cytologically confirmed stage IIIB with malignant pleural or pericardial effusion, stage IV, or recurrent non-squamous NSCLC were eligible. Additional inclusion criteria included at least one measurable target lesion as defined by Response Evaluation Criteria in Solid Tumors (RECIST v1.0); adequate bone marrow, hepatic, and renal function; Eastern Cooperative Oncology Group performance status (ECOG PS) 0 or 1; and no evidence of uncontrolled hypertension (blood pressure [BP] >140/90 mmHg). Antihypertensive medications were allowed. Exclusion criteria included prior systemic therapy for stage IIIB or IV or recurrent NSCLC; prior treatment with a VEGF or VEGF-receptor inhibitor; lung lesion with cavitation, or invading or abutting a major blood vessel; hemoptysis (>2.5 mL in any 24-hr period) <2 weeks before enrollment; National Cancer Institute Common Terminology Criteria for Adverse Events (CTCAE, v3.0) Grade 3 hemorrhage (from any cause) <4 weeks before enrollment; untreated central nervous system metastases; regular use of anticoagulants; or current use or anticipated need for cytochrome P450 (CYP) 3A4-inhibiting or CYP3A4- or CYP1A2-inducing drugs. Each patient provided written informed consent before study entry.

### Study design and treatment

This was a randomized, multicenter, open-label phase II study conducted in 37 centers in 11 countries, and the primary endpoint was PFS assessed by investigators. A non-randomized phase I lead-in (*n* = 10) evaluated the pharmacokinetics and safety of axitinib 5 mg oral dose twice daily (bid) given continuously with pemetrexed 500 mg/m^2^ and cisplatin 75 mg/m^2^ administered once every 21 days [11].

In phase II, eligible patients were stratified by gender and ECOG PS (0 vs. 1) and, using a centralized, randomized permuted block allocation within strata generated by the central randomization administrator, assigned (1:1:1) to receive axitinib bid continuously plus pemetrexed/cisplatin (arm I), axitinib in a modified-dosing schedule plus pemetrexed/cisplatin (arm II), or pemetrexed/cisplatin alone (arm III). Axitinib was administered orally at a starting dose of 5 mg bid in 21-day cycles. For the modified-dosing schedule (arm II), axitinib was given on days 2 through 19, followed by a 3-day interruption (i.e. 2 days before and the day of chemotherapy), except the last cycle, during which it was given on days 2 through 21. Axitinib dose could be increased step-wise to 7 mg bid, and then to a maximum of 10 mg bid, in patients who tolerated axitinib with no treatment-related CTCAE Grade ≥3 AEs for ≥2 weeks, unless BP was greater than 150/90 mmHg or patient was taking antihypertensive medication. Axitinib dose was reduced step-wise to 3 mg bid, and then to 2 mg bid, at the discretion of the investigator, in patients who experienced a treatment-related CTCAE Grade 3 AE or BP >150/100 mmHg on maximal antihypertensive treatment. Axitinib treatment was temporarily interrupted in patients who had a treatment-related CTCAE Grade 4 AE, BP >160/105 mmHg, or urine protein/creatinine ratio ≥2.0 and restarted at the next lower dose once improved to CTCAE Grade ≤2, BP <150/100 mmHg, or urine protein/creatinine ratio <2.0, respectively. If a patient required a dose reduction below 2 mg bid, axitinib was to be discontinued. Pemetrexed 500 mg/m^2^ and cisplatin 75 mg/m^2^ were administered intravenously on day 1 of each of up to six 21-day cycles. Dose reductions were based on nadir hematologic counts or maximum non-hematologic toxicity from the preceding cycle. Vitamin B_12_ (1000 μg) and folic acid (350–1000 μg) were administered ≥1 week prior to treatment and then every 9 weeks and daily, respectively, until 3 weeks after the last dose of chemotherapy.

Patients randomized to arms I and II who completed four to six cycles of axitinib plus pemetrexed/cisplatin and had stable disease or better continued to receive single-agent axitinib maintenance therapy until disease progression, unacceptable toxicity, or withdrawal of patient consent. All patients were followed bimonthly for survival status following discontinuation of study treatment until at least 1 year after randomization of the last patient. Crossover between treatment arms was not allowed.

The study protocol was reviewed and approved by the institutional review board or independent ethics committee at each center. The names of all institutional review boards and independent ethics committees are listed under Appendix. The study was conducted in compliance with the Declaration of Helsinki, International Conference on Harmonization Good Clinical Practice Guidelines, and local regulatory requirements. This trial was registered at ClinicalTrials.gov (NCT00768755) on October 7, 2008.

### Assessments

Radiologic tumor assessments were performed at screening and every 6 weeks thereafter, and whenever disease progression was suspected. Responses were evaluated according to RECIST and required confirmation ≥4 weeks after initial documentation. Safety was evaluated throughout the study. BP measurements were taken at screening and on day 1 of each cycle and thyroid function tests were conducted at screening and on day 1 of each chemotherapy cycle (cycles 1–6) and on day 1 of every other cycle thereafter. In addition, patients in arms I and II self-monitored BP bid at home prior to axitinib dosing and were instructed to contact their physicians for further evaluation of systolic BP >150 mmHg or diastolic BP >100 mmHg. Patient-reported outcomes (PROs) were evaluated, using the M. D. Anderson Symptom Inventory (MDASI) questionnaire on days 1 and 8 of each chemotherapy cycle and on day 1 of each axitinib maintenance cycle. MDSAI is a 19-item, validated self-reported questionnaire consisting of two scales that assess symptom severity and interference with different aspects of patient’s life [12]. Mean change in the MDASI score ≥0.98 point was defined as clinically meaningful.

### Statistical analysis

The primary purpose of this study was to assess the efficacy (as measured by PFS) of axitinib in combination with pemetrexed/cisplatin versus pemetrexed/cisplatin alone in patients with non-squamous NSCLC in the randomized phase II study. The sample size estimates were based on separate comparisons of the axitinib-containing arms I and II versus arm III (pemetrexed/cisplatin alone). Fifty patients were required in each arm and 70 events for each comparison for a two-sample log-rank test to have an overall one-sided significance level of 0.20 and power of 0.80. This assumed a 50% improvement in median PFS from 5.0 months in arm III to 7.5 months in arm I or II, and ~12-month accrual time and 6-month follow-up. The hazard ratio and its 95% CI were estimated. A stratified log-rank test (one-sided, α = 0.20) was used to compare PFS between the treatment arms; however, the *P* values were for reference only.

Secondary endpoints included OS, ORR, duration of tumor response, PROs, and safety. ORR between treatment arms was compared using Cochran-Mantel-Haenszel test stratified by baseline ECOG PS and gender. Descriptive summary statistics (mean with standard deviation of absolute scores and mean change from baseline with 95% CI) of the MDASI items were reported. Safety was analyzed in patients who received at least one dose of study drug, and the results from only the randomized phase II portion were presented here.

The efficacy and safety analyses were originally conducted based on the data obtained as of March 1, 2011, while the study was still ongoing. PFS and overall safety were later updated using a data cutoff date of December 21, 2011, which are presented here. It should be noted that median PFS in each arm were very similar between the two analyses. The final analysis for OS, duration of tumor response among responders, number of deaths, and serious AEs was conducted after the database lock on May 18, 2012. For each endpoint, the most-up-to date results are presented in this manuscript.

## Results

### Patient characteristics

**Figure 1:**
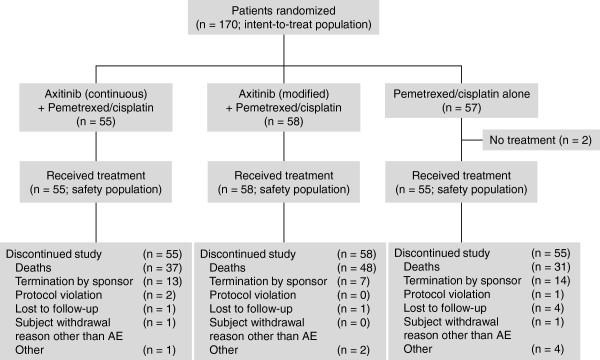
**Summary of patient disposition.** AE, adverse event.

Between January 19, 2009 and April 21, 2010, a total of 170 patients were randomly assigned among three treatment arms: arm I (*n* = 55), arm II (*n* = 58), and arm III (*n* = 57; Figure 1). All patients were treated with assigned drugs, except two patients in arm III who did not receive pemetrexed/cisplatin. Among patients across the three treatment arms, the median age was similar (Table 1). The majority of patients were white (range, 71–84%) and male (range, 62–65%), and diagnosed with stage IV NSCLC (range, 84–91%). Smokers (both current and former) comprised 73%, 84%, and 79% of patients in arms I, II, and III, respectively.

**Table 1:** Baseline patient demographics and clinical characteristics

**Demographics or Clinical characteristics**	**Arm I: Axitinib (Continuous) + Pem/Cis**	**Arm II: Axitinib (Modified) + Pem/Cis**	**Arm III: Pem/Cis Alone**
	***n *** **= 55**	***n *** **= 58**	***n *** **= 57**
Age, yr, median (range)	62 (30–77)	62 (35–83)	59 (42–76)
Gender, *n* (%)			
Male	34 (62)	37 (64)	37 (65)
Female	21 (38)	21 (36)	20 (35)
Race, *n* (%)			
White	39 (71)	49 (84)	45 (79)
Black	0	1 (2)	0
Asian	15 (27)	8 (14)	12 (21)
Other	1 (2)	0	0
Smoking status, *n* (%)			
Never smoked	15 (27)	9 (16)	12 (21)
Smoker^a^	40 (73)	49 (84)	45 (79)
ECOG PS, *n* (%)			
0	25 (45)	25 (43)	27 (47)
1	30 (55)	33 (57)	28 (49)
Not reported	0	0	2 (4)
Histological classification, *n* (%)			
Adenocarcinoma	53 (96)	52 (90)	47 (82)
Large cell	1 (2)	3 (5)	8 (14)
Bronchioloalveolar	0	2 (3)	2 (4)
Other	1 (2)	1 (2)	0
Disease stage at baseline, *n* (%)			
Stage IIIB	3 (5)	6 (10)	4 (7)
Stage IV	46 (84)	50 (86)	52 (91)
Recurrent	6 (11)	2 (3)	1 (2)
Prior therapy, *n* (%)			
Resection^b^	14 (25)	12 (21)	16 (28)
Radiation	11 (20)	7 (12)	8 (14)

*Abbreviations: Pem/Cis* Pemetrexed/cisplatin, *ECOG PS* Eastern Cooperative Oncology Group performance status.

^a^Included both active and ex-smokers.

^b^Included partial resection.

### Treatment

The median number of cycles for pemetrexed and cisplatin was similar across all treatment arms: five cycles each in arm I, six and five cycles, respectively, in arm II, and six cycles each in arm III. The median (range) of axitinib treatment cycles was 8 (1–28) in arm I and 6.5 (1–22) in arm II. Patients in arm I received axitinib treatment longer than those in arm II (median days on axitinib: 158 and 117 days, respectively). One or more axitinib dose interruptions were reported in 87% of patients in arm I and 97% in arm II, of which 76% and 69%, respectively, were due to AEs. Median relative axitinib dose intensity (defined as [total dose administered/total dose assigned] × 100) was 92% in arm I and 104% in arm II. Median relative dose intensity was similar between the three arms for pemetrexed (99%, 99%, and 100%) and for cisplatin (98%, 99%, and 100%). Following combination treatment, 58% of patients in arm I and 50% in arm II received single-agent axitinib maintenance therapy. By the completion of the study, all patients discontinued the study, mostly due to death (*n* = 116; Figure 1).

### Efficacy

**Figure 2:**
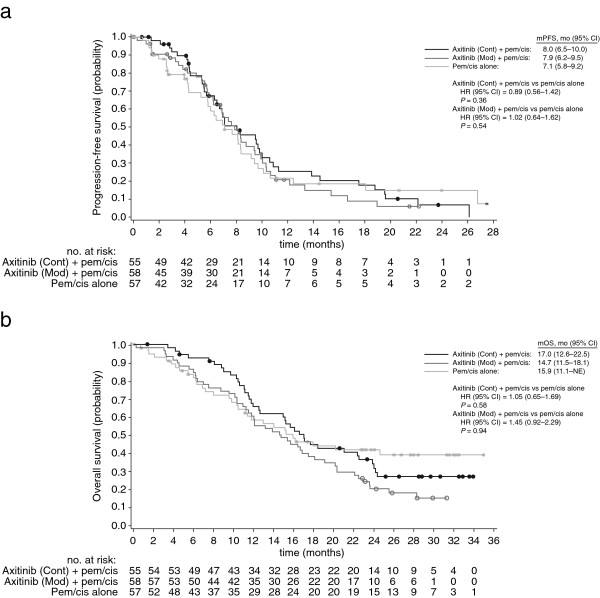
**Kaplan-Meier estimates for (a) progression-free survival and (b) overall survival.***P* values were based on one-sided log-rank test stratified by Eastern Cooperative Oncology Group performance status and gender. Progression-free survival was based on data cutoff date of December 21, 2011 and overall survival was based on the most recent data at the time of final database lock on May 18, 2012. CI, confidence interval; Cont, continuously; HR, hazard ratio; mod, modified schedule; mOS, median overall survival; mPFS, median progression-free survival.

The investigator-assessed median (95% CI) PFS was 8.0 (6.5–10.0), 7.9 (6.2–9.5), and 7.1 (5.8–9.2) months in arms I, II, and III, respectively (Figure 2A). The hazard ratio (95% CI) was 0.89 (0.56–1.42; *P* = 0.36) for arm I versus arm III, and 1.02 (0.64–1.62; *P* = 0.54) for arm II versus arm III. Median OS (95% CI) was 17.0 (12.6–22.5), 14.7 (11.5–18.1), and 15.9 (11.1–not estimable) months in arms I, II, and III, respectively (Figure 2B). Overall confirmed ORRs (95% CI) was 45.5% (32.0–59.4) and 39.7% (27.0–53.4) for the axitinib-containing arms I and II, respectively, which were both higher than the 26.3% (15.5–39.7) in arm III (Table 2). Median (95% CI) duration of tumor response among responders was 7.8 (5.6–11.4), 6.7 (5.0–7.8), and 7.1 (4.2–24.7) months in arms I (*n* = 25), II (*n* = 23), and III (*n* = 15), respectively.

**Table 2:** **Investigator-assessed best tumor response**^**a**^

	**Arm I: Axitinib (Continuous) + Pem/Cis**	**Arm II: Axitinib (Modified) + Pem/Cis**	**Arm III: Pem/Cis Alone**
	***n *** **= 55**^**b**^	***n *** **= 58**	***n *** **= 57**^**b**^
Best overall response, *n* (%)			
CR	0	0	0
PR	25 (45.5)	23 (39.7)	15 (26.3)
SD (≥8 weeks)	18 (32.7)	19 (32.8)	22 (38.6)
PD	4 (7.3)	7 (12.1)	8 (14.0)
Not assessed	0	1 (1.7)	1 (1.8)
Indeterminate^c^	7 (12.7)	8 (13.8)	9 (15.8)
Overall confirmed ORR, *n* (%)	25 (45.5)	23 (39.7)	15 (26.3)
95% CI	32.0–59.4	27.0–53.4	15.5–39.7
Treatment comparison, risk ratio^d^ (95% CI) vs. arm III	1.75	1.51	–
	(1.05–2.94)	(0.87–2.63)	–
*P*^e^	0.01	0.07	–

*Abbreviations: Pem/Cis* Pemetrexed/cisplatin, *CR* Complete response, *PR* Partial response, *SD* Stable disease, *PD* Progressive disease, *ORR* Objective response rate, *CI* Confidence interval.

^a^Based on data cutoff date of March 11, 2011.

^b^One patient in arm I and two patients in arm III had no measureable disease at baseline.

^c^Patients who did not have evaluable baseline scan or no post-randomization scan or those who had stable disease for <8 weeks.

^d^Calculated based on a normal distribution.

^e^One-sided Cochran-Mantel-Haenszel test stratified by Eastern Cooperative Oncology Group performance status and gender.

### Safety

Gastrointestinal disorders (nausea, vomiting, decreased appetite, and constipation) and fatigue were common treatment-emergent, all-causality AEs in all three treatment arms (Table 3). Hypertension, diarrhea, and dysphonia occurred more frequently in axitinib-containing arms compared with pemetrexed/cisplatin alone. The most common Grade 3 AEs were hypertension in axitinib-containing arms (20% in arm I and 17% in arm II) and fatigue with pemetrexed/cisplatin alone (16%). Asthenia and pulmonary embolism were the only Grade 4 AEs observed in more than one patient in any arm (*n* = 2 each, arm II). Serious AEs reported by more than three patients in any arm were vomiting, nausea, and dehydration.

**Table 3:** **Treatment-emergent, all-causality adverse events and laboratory abnormalities in ≥20% of patients in any treatment arm**^**a**^

**Adverse events, *n *** **(%)**	**Arm I: Axitinib (Continuous) + Pem/Cis**		**Arm II: Axitinib (Modified) + Pem/Cis**		**Arm III: Pem/Cis Alone**	
	**All**	**Grade**	**All**	**Grade**	**All**	**Grade**
	**Grades**	**3/4**	**Grades**	**3/4**	**Grades**	**3/4**
	***n *** **= 55**	***n *** **= 55**	***n *** **= 58**	***n *** **= 58**	***n *** **= 55**	***n *** **= 55**
Nausea	38 (69)	10 (18)	30 (52)	3 (5)	33 (60)	4 (7)
Hypertension	37 (67)	11 (20)	28 (48)	10 (17)	6 (11)	0
Vomiting	29 (53)	8 (15)	19 (33)	3 (5)	16 (29)	2 (4)
Decreased appetite	28 (51)	4 (7)	28 (48)	4 (7)	23 (42)	2 (4)
Fatigue	27 (49)	6 (11)	25 (43)	9 (16)	25 (45)	9 (16)
Diarrhea	21 (38)	4 (7)	20 (34)	3 (5)	9 (16)	0
Constipation	20 (36)	0	15 (26)	1 (2)	23 (42)	1 (2)
Neutropenia	17 (31)	7 (13)	12 (21)	6 (10)	15 (27)	5 (9)
Dyspnea	13 (24)	3 (5)	14 (24)	2 (3)	10 (18)	3 (5)
Insomnia	13 (24)	0	8 (14)	1 (2)	11 (20)	0
Dysphonia	12 (22)	0	8 (14)	0	0	0
Cough	11 (20)	1 (2)	7 (12)	0	9 (16)	2 (4)
Headache	11 (20)	0	17 (29)	1 (2)	9 (16)	0
Anemia	7 (13)	3 (5)	20 (34)	8 (14)	23 (42)	6 (11)
**Laboratory abnormalities, *n*/*N***^**b **^**(%)**						
Hyperglycemia	40/53 (75)	1/53 (2)	36/55 (65)	3/55 (5)	33/52 (63)	2/52 (4)
Hypoalbuminemia	26/50 (52)	1/50 (2)	31/54 (57)	1/54 (2)	23/50 (46)	0
Hypokalemia	23/53 (43)	0	17/55 (31)	1/55 (2)	10/52 (19)	0
Creatinine	22/54 (41)	2/54 (4)	29/55 (53)	5/55 (9)	24/53 (45)	3/53 (6)
Hyponatremia	18/54 (33)	3/54 (6)	18/55 (33)	7/55 (13)	11/52 (21)	1/52 (2)
Alkaline phosphatase	16/53 (30)	0	23/55 (42)	0	15/53 (28)	0
Hyperkalemia	16/53 (30)	2/53 (4)	26/55 (47)	2/55 (4)	19/52 (37)	2/52 (4)
ALT	15/53 (28)	1/53 (2)	23/55 (42)	1/55 (2)	12/53 (23)	2/53 (4)
Bicarbonate	8/30 (27)	0	17/38 (45)	1/38 (3)	13/39 (33)	0
AST	10/53 (19)	1/53 (2)	21/55 (38)	1/55 (2)	10/53 (19)	2/53 (4)

*Abbreviations*: *Pem/Cis* Pemetrexed/cisplatin, *ALT* Alanine aminotransferase, *AST* Aspartate aminotransferase.

^a^Based on data cutoff date of December 21, 2011.

^b^Denominator for each laboratory abnormality differed depending on the availability of baseline and at least one test result during the study treatment.

The majority of laboratory abnormalities reported during the study were Grade 1 or 2. Abnormal neutrophil count was the most common Grade 3/4 laboratory abnormality among all three treatment arms (Table 3). Hypothyroidism was reported infrequently (≤5%) in axitinib-containing arms, and no severe hemorrhagic events occurred in any treatment arm.

### Patient-reported outcomes

At baseline, mean MDASI symptom severity (13-item summary) and interference scores (6-item summary) were similar among treatment arms (mean severity scores, 1.75, 2.09, and 1.80 and mean interference scores, 2.36, 2.97, and 2.64 in arms I, II, and III, respectively). Overall, there were statistical increases in both mean symptom severity and interference scores compared with baseline, indicating some clinically meaningful worsening of symptom severity and interference with patient feeling and function, in all three treatment arms. However, the majority of absolute symptom severity and interference scores remained <3.0 on a scale of 0 to 10.

## Discussion

This study showed that axitinib, a selective antiangiogenic TKI targeting VEGF receptors, in combination with pemetrexed/cisplatin was generally well tolerated in patients with advanced non-squamous NSCLC. However, the study did not achieve its primary endpoint (PFS), irrespective of axitinib continuous or intermittent-dosing schedules. In addition, although combination therapy resulted in numerically higher ORR than chemotherapy alone, it did not improve OS.

While cross-study comparison is complicated due to many variables, median PFS and OS in patients treated with pemetrexed/cisplatin alone in this study were longer than the 4.8 and 10.3 months, respectively, observed in a prior large phase III trial of pemetrexed/cisplatin in chemotherapy-naïve NSCLC patients [10]. One plausible explanation is the selection of patients with non-squamous histology in the current study. Compared with the previous study [10], this study also had a higher percentage of Asians (21% vs. 13%), non-smokers (21% vs. 15%), and patients with ECOG PS 0 (47% vs. 35%), all of which have been identified as prognostic factors in advanced NSCLC [13]. Another possible explanation for longer survival in the control arm may be due to the subsequent therapies. Although the percentage of patients in this study who received any follow-up systemic therapy post-study, including EGFR inhibitors, was not too different from that reported for patients who received pemetrexed/cisplatin in the previous phase III trial [10] (47% compared with 52.6%, respectively), no data were available in either study to identify individuals with genomic mutations in *EGFR* or *ALK*, who would have benefited from the specific molecularly-targeted follow-up therapy. It should also be noted that clinical outcomes in a phase II study with a small number of patients do not always reflect the results of a subsequent phase III study, as seen with other agents.

Since the Sandler et al. [6] landmark study demonstrated significant survival benefits of adding bevacizumab to platinum doublet chemotherapy, several antiangiogenic TKIs have been evaluated in combination with cytotoxic agents, but with generally disappointing results [14-16]. In randomized phase III trials, addition of sorafenib to either paclitaxel/carboplatin in chemotherapy-naïve patients with advanced NSCLC [14] or gemcitabine/cisplatin in advanced non-squamous NSCLC [16] did not meet the primary endpoint of OS. In another recent phase III trial, combination therapy with motesanib, another antiangiogenic TKI, plus paclitaxel/carboplatin also failed to prolong OS [15]. The current study of axitinib in combination with pemetrexed/cisplatin adds to a growing list of antiangiogenic TKIs that do not provide significant survival benefits when combined with standard doublet chemotherapy in advanced NSCLC, albeit with acceptable toxicity.

Reasons for apparent failure of antiangiogenic TKIs to improve efficacy of conventional chemotherapy are unclear, but are likely multifactorial and may include timing of administering antiangiogenic agents relative to cytotoxic agents, as well as off-target activities of antiangiogenic TKIs, adding to the toxicity. The potency of TKIs in inhibiting VEGF receptors determined in vitro may not necessarily translate to better efficacy in combination with cytotoxic agents. It is postulated that bevacizumab induces normalization of the tumor vasculature, thereby facilitating uptake of cytotoxic agents [17,18]. In contrast, combination axitinib plus cyclophosphamide resulted in decreased tumor uptake of activated cyclophosphamide (4-hydroperoxy-cyclophosphamide) and decreased antitumor efficacy in a preclinical study [19]. Based on [^18^F]fluorodeoxythymidine (FLT) positron emission tomography/computed tomography imaging, continuous administration of axitinib in patients with advanced solid tumors appears to reduce the tumor uptake of FLT, which is reverted to baseline following axitinib dosing interruption [20,21]. Reduced FLT uptake could indicate decreased tumor proliferation, but also decreased cytotoxic drug delivery to the tumor, which would reduce the activity of cytotoxic agents. In the current study, it was hoped that stopping axitinib administration 2 days before and on the day of chemotherapy would alleviate the latter effect of axitinib, but no improvement in efficacy was observed. Clearly, there is an urgent need for better understanding of the complex nature of tumor angiogenesis and how axitinib and other antiangiogenic TKIs affect not only the tumor vasculature but also various cellular components within the tumor microenvironment [22].

With regard to toxicity, addition of axitinib to standard doses of pemetrexed and cisplatin did not lead to AEs that were unexpected, based on studies with single-agent axitinib [8] or pemetrexed/cisplatin alone [10] in advanced NSCLC. Compared with chemotherapy alone, incidence of hypertension increased substantially in patients receiving axitinib-containing treatment, which has been observed with antiangiogenic agents in general [16,23,24]. In the current axitinib-containing arms, no severe hemorrhagic incidence was reported.

Therefore, axitinib in combination with pemetrexed/cisplatin was generally tolerable and AEs were manageable in patients with advanced non-squamous NSCLC. Addition of axitinib resulted in numerically higher ORR, but did not improve PFS or OS compared with chemotherapy alone. However, it remains to be seen if certain subsets of patients may derive some benefits from the use of TKIs, including axitinib, as reported for other TKIs in patients with genomic abnormalities such as *EGFR* mutations [25-27], crizotinib in ALK-positive NSCLC [28], or in preclinical studies involving *RET* proto-oncogene rearrangements [29,30].

## Conclusions

In patients with advanced non-squamous NSCLC, axitinib in combination with pemetrexed plus cisplatin was generally well tolerated and resulted in numerically higher ORR compared with chemotherapy alone. However, addition of axitinib — continuous dosing or with a 3-day break around the time of chemotherapy — did not improve PFS (primary endpoint) or OS over chemotherapy alone.

## Appendix

The names of all institutional review boards and independent ethics committees were: Comitato Etico Azienda Ospedaliera Universitaria San Luigi Gonzaga di Orbassano (Orbassano, Italy); Comitato Etico dell’IRCCS Istituto Nazionale per la Ricerca sul Cancro di Genova (Genova, Italy); Comitato Etico Locale per la Sperimentazione Clinica della AUSL 12 di Viareggio (Camaiore, Italy); Shizuoka Cancer Center Institutional Review Board (Shizuoka, Japan); Komisja Bioetyczna przy Okregowej Izbie Lekarskiej w Gdansku (Gdansk, Poland); Academia de Stiinte Medicale, Comisia Nationala de Etica pentru Studiul Clinic al Medicamentului (Bucuresti, Romania); Ethics Committee at the Federal Service on Surveillance in Healthcare and Social Development (Moscow, Russian Federation); Ethics Committee of RUSSIAN ONCOLOGICAL RESEARCH CENTER n.a. N.N. BLOKHIN RAMS (Moscow; Russian Federation); Ethics Committee Saint-Petersburg State Medical University named after I.P. Pavlov of Roszdrav (Saint Petersburg, Russian Federation); Ethics Council at the Ministry of Healthcare and Social Development of Russian Federation (Moscow, Russian Federation); Ethics Committee of the Medical Military Academy named after S.M. Kirov (Saint Petersburg, Russian Federation); Local Ethics Committee of the Pyatigorsk Oncology Center (Pyatigorsk, Russian Federation); University of the Witwatersrand Human Research Ethics Committee (Medical) (Johannesburg, South Africa); Hospital General Universitario Gregorio Marañon Ethics Committee of Clinical Investigation (Madrid, Spain): Ethikkommission beider Basel EKBB (Basel, Switzerland); Comitato Etico Cantonale c/o Sezione sanitaria (Bellinzona, Switzerland); Veterans General Hospital-Taipei Institutional Review Board Medical Research and Education (Taipei, Taiwan); Chung Shan Medical University Hospital Institutional Review Board (Taichung, Taiwan); National Taiwan University Hospital Research Ethics Committee (Taipei, Taiwan); Taichung Veterans General Hospital Institutional Review Board (Taichung, Taiwan); Central Committee for Ethics Issues of Ministry of Health of Ukraine (Kyiv, Ukraine); Local Committee for Ethics Issues of Kyiv City Clinical Oncologic Center (Kyiv, Ukraine); Committee for Ethics Issues at Dnipropetrovsk City Multiple-Discipline Clinical Hospital #4 (Dnipropetrovsk, Ukraine); Commission for Ethics Issues of Cherkasy Regional Oncology Dispensary (Cherkasy, Ukraine); South West - Exeter South West Research Ethics Committee Centre (Bristol, UK); Schulman Associates Institutional Review Board Incorporated (Cincinnati, OH, USA); Southern Illinois University School of Medicine Springfield Committee for Research Involving Human Subjects (SCRIHS) (Springfield, IL, USA); Penn State College of Medicine, Penn State Milton S. Hershey Medical Center Institutional Review Board (Hershey, PA, USA); Peoria Institutional Review Board (Peoria, IL, USA).

## Abbreviations

AE: Adverse event; ALK: Anaplastic lymphoma kinase; bid: Twice daily; BP: Blood pressure; CI: Confidence interval; CTCAE: Common Terminology Criteria for Adverse Events; CYP: Cytochrome P450; ECOG PS: Eastern Cooperative Oncology Group performance status; EGFR: Epidermal growth factor receptor; FLT: [^18^F] fluorodeoxythymidine; MDASI: M. D. Anderson Symptom Inventory; NSCLC: Non-small-cell lung cancer; ORR: Objective response rate; OS: Overall survival; PFS: Progression-free survival; PROs: Patient-reported outcomes; RECIST: Response Evaluation Criteria in Solid Tumors; TKI: Tyrosine kinase inhibitor; VEGF: Vascular endothelial growth factor.

## Competing interests

CPB, NY, IMB, AP, and SN declare no relevant financial conflicts of interest. JT, PB, AGN, and AI are employees of and own stock in Pfizer Inc. SK, employed at Pfizer Inc at the time of the study described here and development of this manuscript is currently employed by Mirna Therapeutics and owns stock in Pfizer Inc and Mirna Therapeutics. GVS received honoraria from Eli Lilly, Roche, AstraZeneca, and Pfizer Inc.

## Authors’ contributions

CPB, PB, AGN, and SK contributed to the conception and design of the study. NY, IMB, AP, AGN, SK, and GVS collected and assembled data. SN, JT, PB, AGN, AI, SK, and GVS undertook the data analysis and interpretation. All authors participated in the development of the manuscript and approved the final manuscript.

## Authors’ information

Sinil Kim was employed at Pfizer Inc at the time of the study described here and development of this manuscript.

## Pre-publication history

The pre-publication history for this paper can be accessed here:

http://www.biomedcentral.com/1471-2407/14/290/prepub

## References

[B1] Cancer facts & figures 2012. Atlanta: American Cancer Society, 2012 [http://www.cancer.org/acs/groups/content/@epidemiologysurveilance/documents/document/acspc-031941.pdf]

[B2] Wang T Nelson RA Bogardus A Grannis FW Jr Five-year lung cancer survival: which advanced stage nonsmall cell lung cancer patients attain long-term survival? Cancer 2010 116 1518 1525 10.1002/cncr.24871 20108308

[B3] Schiller JH Harrington D Belani CP Langer C Sandler A Krook J Zhu J Johnson DH Eastern Cooperative Oncology Group Comparison of four chemotherapy regimens for advanced non-small-cell lung cancer N Engl J Med 2002 346 92 98 10.1056/NEJMoa011954 11784875

[B4] Xiao YY Zhan P Yuan DM Liu HB Lv TF Song Y Shi Y Chemotherapy plus multitargeted antiangiogenic tyrosine kinase inhibitors or chemotherapy alone in advanced NSCLC: a meta-analysis of randomized controlled trials Eur J Clin Pharmacol 2013 69 151 159 10.1007/s00228-012-1333-3 22729611

[B5] Ellis PM Al-Saleh K Multitargeted anti-angiogenic agents and NSCLC: clinical update and future directions Crit Rev Oncol Hematol 2012 84 47 58 10.1016/j.critrevonc.2012.02.004 22405734

[B6] Sandler A Gray R Perry MC Brahmer J Schiller JH Dowlati A Lilenbaum R Johnson DH Paclitaxel-carboplatin alone or with bevacizumab for non-small-cell lung cancer N Engl J Med 2006 355 2542 2550 10.1056/NEJMoa061884 17167137

[B7] Hu-Lowe DD Zou HY Grazzini ML Hallin ME Wickman GR Amundson K Chen JH Rewolinski DA Yamazaki S Wu EY McTigue MA Murray BW Kania RS O’Connor P Shalinsky DR Bender SL Nonclinical antiangiogenesis and antitumor activities of axitinib (AG-013736), an oral, potent, and selective inhibitor of vascular endothelial growth factor receptor tyrosine kinases 1, 2, 3 Clin Cancer Res 2008 14 7272 7283 10.1158/1078-0432.CCR-08-0652 19010843

[B8] Schiller JH Larson T Ou SH Limentani S Sandler A Vokes E Kim S Liau K Bycott P Olszanski AJ von Pawel J Efficacy and safety of axitinib in patients with advanced non-small-cell lung cancer: results from a phase II study J Clin Oncol 2009 27 3836 3841 10.1200/JCO.2008.20.8355 19597027

[B9] Kozloff MF Martin LP Krzakowski M Samuel TA Rado TA Arriola E De Castro Carpeño J Herbst RS Tarazi J Kim S Rosbrook B Tortorici M Olszanski AJ Cohen RB Phase I trial of axitinib combined with platinum doublets in patients with advanced non-small cell lung cancer and other solid tumours Br J Cancer 2012 107 1277 1285 10.1038/bjc.2012.406 22990652 PMC3494447

[B10] Scagliotti GV Parikh P von Pawel J Biesma B Vansteenkiste J Manegold C Serwatowski P Gatzemeier U Digumarti R Zukin M Lee JS Mellemgaard A Park K Patil S Rolski J Goksel T de Marinis F Simms L Sugarman KP Gandara D Phase III study comparing cisplatin plus gemcitabine with cisplatin plus pemetrexed in chemotherapy-naive patients with advanced-stage non-small-cell lung cancer J Clin Oncol 2008 26 3543 3551 10.1200/JCO.2007.15.0375 18506025

[B11] Tortorici MA Iglesias L Kozloff MF Pithavala YK Ingrosso A Belani CP Pharmacokinetic (PK) analysis of coadministration of axitinib and pemetrexed/cisplatin (pem/cis) in patients with non–small cell lung cancer (NSCLC) [abstract PI-69] Clin Pharmacol Ther 2012 91 Suppl 1 S33

[B12] Cleeland CS The M. D. Anderson Symptom Inventory User Guide [Draft]. Houston, Texas, University of Texas M. D. Anderson Cancer Center 2010 Houston, TX: The University of Texas MD Anderson Cancer Center [http://www.mdanderson.org/education-and-research/departments-programs-and-labs/departments-and-divisions/symptom-research/symptom-assessment-tools/MDASI_userguide.pdf]

[B13] Pirker R Pereira JR Szczesna A von Pawel J Krzakowski M Ramlau R Vynnychenko I Park K Eberhardt WE de Marinis F Heeger S Goddemeier T O’Byrne KJ Gatzemeier U Prognostic factors in patients with advanced non-small cell lung cancer: data from the phase III FLEX study Lung Cancer 2012 77 376 382 10.1016/j.lungcan.2012.03.010 22498112

[B14] Scagliotti G Novello S von Pawel J Reck M Pereira JR Thomas M Abrao Miziara JE Balint B de Marinis F Keller A Aren O Csollak M Albert I Barrios CH Grossi F Krzakowski M Cupit L Cihon F Dimatteo S Hanna N Phase III study of carboplatin and paclitaxel alone or with sorafenib in advanced non-small-cell lung cancer J Clin Oncol 2010 28 1835 1842 10.1200/JCO.2009.26.1321 20212250

[B15] Scagliotti GV Vynnychenko I Park K Ichinose Y Kubota K Blackhall F Pirker R Galiulin R Ciuleanu TE Sydorenko O Dediu M Papai-Szekely Z Banaclocha NM McCoy S Yao B Hei YJ Galimi F Spigel DR International, randomized, placebo-controlled, double-blind phase III study of motesanib plus carboplatin/paclitaxel in patients with advanced non-squamous non-small-cell lung cancer: MONET1 J Clin Oncol 2012 30 2829 2836 10.1200/JCO.2011.41.4987 22753922

[B16] Paz-Ares LG Biesma B Heigener D von Pawel J Eisen T Bennouna J Zhang L Liao M Sun Y Gans S Syrigos K Le Marie E Gottfried M Vansteenkiste J Alberola V Strauss UP Montegriffo E Ong TJ Santoro A NSCLC [non-small-cell lung cancer] Research Experience Utilizing Sorafenib (NExUS) Investigators Study Group Phase III, randomized, double-blind, placebo-controlled trial of gemcitabine/cisplatin alone or with sorafenib for the first-line treatment of advanced, non-squamous non-small-cell lung cancer J Clin Oncol 2012 30 3084 3092 10.1200/JCO.2011.39.7646 22851564

[B17] Dickson PV Hamner JB Sims TL Fraga CH Ng CY Rajasekeran S Hagedorn NL McCarville MB Stewart CF Davidoff AM Bevacizumab-induced transient remodeling of the vasculature in neuroblastoma xenografts results in improved delivery and efficacy of systemically administered chemotherapy Clin Cancer Res 2007 13 3942 3950 10.1158/1078-0432.CCR-07-0278 17606728

[B18] Goel S Duda DG Xu L Munn LL Boucher Y Fukumura D Jain RK Normalization of the vasculature for treatment of cancer and other diseases Physiol Rev 2011 91 1071 1121 10.1152/physrev.00038.2010 21742796 PMC3258432

[B19] Ma J Waxman DJ Modulation of the antitumor activity of metronomic cyclophosphamide by the angiogenesis inhibitor axitinib Mol Cancer Ther 2008 7 79 89 18202011 10.1158/1535-7163.MCT-07-0584 PMC2390754

[B20] Hoh C Infante JR Burris HA Tarazi JC Kim S Rosbrook B Reid TR Axitinib inhibition of [^18^F] fluorothymidine (FLT) uptake in patients (pts) with colorectal cancer (CRC): implications for cytotoxic chemotherapy combinations [abstract 3591] J Clin Oncol 2011 29 Suppl 15s

[B21] Jeraj R Liu G Simoncic U Vanderhoek M Perlman S Alberti DB Harrison MR Wilding G Concurrent assessment of vasculature and proliferative pharmacodynamics in patients treated with VEGFR TKI [abstract 3050] J Clin Oncol 2010 28 Suppl 15s

[B22] Joyce JA Therapeutic targeting of the tumor microenvironment Cancer Cell 2005 7 513 520 10.1016/j.ccr.2005.05.024 15950901

[B23] Dahlberg SE Sandler AB Brahmer JR Schiller JH Johnson DH Clinical course of advanced non-small-cell lung cancer patients experiencing hypertension during treatment with bevacizumab in combination with carboplatin and paclitaxel on ECOG 4599 J Clin Oncol 2010 28 949 954 10.1200/JCO.2009.25.4482 20085937 PMC2834434

[B24] Rini BI Schiller JH Fruehauf JP Cohen EE Tarazi JC Rosbrook B Bair AH Ricart AD Olszanski AJ Letrent KJ Kim S Rixe O Diastolic blood pressure as a biomarker of axitinib efficacy in solid tumors Clin Cancer Res 2011 17 3841 3849 10.1158/1078-0432.CCR-10-2806 21531811

[B25] Mok TSK Paz-Ares L Wu Y-L Novello S Juhasz E Aren O Sun Y Hirsh V Smit EF Lathia C Ong TJ Pena C Association between tumor EGFR and KRas mutation status and clinical outcomes in NSCLC patients randomized to sorafenib plus best supportive care (BSC) or BSC alone: subanalysis of the phase III MISSION trial [abstract LBA9_PR] Ann Oncol 2012 23 Suppl 9 ixe1

[B26] Paez JG Janne PA Lee JC Tracy S Greulich H Gabriel S Herman P Kaye FJ Lindeman N Boggon TJ Naoki K Sasaki H Fujii Y Eck MJ Sellers WR Johnson BE Meyerson M EGFR mutations in lung cancer: correlation with clinical response to gefitinib therapy Science 2004 304 1497 1500 10.1126/science.1099314 15118125

[B27] Cappuzzo F Ciuleanu T Stelmakh L Cicenas S Szczesna A Juhasz E Esteban E Molinier O Brugger W Melezinek I Klingelschmitt G Klughammer B Giaccone G SATURN investigators Erlotinib as maintenance treatment in advanced non-small-cell lung cancer: a multicentre, randomised, placebo-controlled phase 3 study Lancet Oncol 2010 11 521 529 10.1016/S1470-2045(10)70112-1 20493771

[B28] Kwak EL Bang YJ Camidge DR Shaw AT Solomon B Maki RG Ou SH Dezube BJ Janne PA Costa DB Varella-Garcia M Kim WH Lynch TJ Fidias P Stubbs H Engelman JA Sequist LV Tan W Gandhi L Mino-Kenudson M Wei GC Shreeve SM Ratain MJ Settleman J Christensen JG Haber DA Wilner K Salgia R Shapiro GI Clark JW Anaplastic lymphoma kinase inhibition in non-small-cell lung cancer N Engl J Med 2010 363 1693 1703 10.1056/NEJMoa1006448 20979469 PMC3014291

[B29] Jeong WJ Mo JH Park MW Choi IJ An SY Jeon EH Ahn SH Sunitinib inhibits papillary thyroid carcinoma with RET/PTC rearrangement but not BRAF mutation Cancer Biol Ther 2011 12 458 465 10.4161/cbt.12.5.16303 21725210

[B30] Henderson YC Ahn SH Kang Y Clayman GL Sorafenib potently inhibits papillary thyroid carcinomas harboring RET/PTC1 rearrangement Clin Cancer Res 2008 14 4908 4914 10.1158/1078-0432.CCR-07-1772 18676765 PMC4420193

